# Secondary Outcomes of a Pilot Randomized Trial of Azithromycin Treatment for Asthma

**DOI:** 10.1371/journal.pctr.0010011

**Published:** 2006-06-30

**Authors:** David L Hahn, Mary Beth Plane, Olaimatu S Mahdi, Gerald I Byrne

**Affiliations:** 1 Dean Medical Center, East Clinic, Madison, Wisconsin, United States of America; 2 Department of Family Medicine, University of Wisconsin Medical School, Madison, Wisconsin, United States of America; 3 Department of Molecular Sciences, University of Tennessee Health Science Center, Memphis, Tennessee, United States of America

## Abstract

**Objectives:**

The respiratory pathogen *Chlamydia pneumoniae (C. pneumoniae)* produces acute and chronic lung infections and is associated with asthma. Evidence for effectiveness of antichlamydial antibiotics in asthma is limited. The primary objective of this pilot study was to investigate the feasibility of performing an asthma clinical trial in practice settings where most asthma is encountered and managed. The secondary objectives were to investigate (1) whether azithromycin treatment would affect any asthma outcomes and (2) whether C. pneumoniae serology would be related to outcomes. This report presents the secondary results.

**Design:**

Randomized, placebo-controlled, blinded (participants, physicians, study personnel, data analysts), allocation-concealed parallel group clinical trial.

**Setting:**

Community-based health-care settings located in four states and one Canadian province.

**Participants:**

Adults with stable, persistent asthma.

**Interventions:**

Azithromycin (six weekly doses) or identical matching placebo, plus usual community care.

**Outcome Measures:**

Juniper Asthma Quality of Life Questionnaire (Juniper AQLQ), symptom, and medication changes from baseline (pretreatment) to 3 mo posttreatment (follow-up); C. pneumoniae IgG and IgA antibodies at baseline and follow-up.

**Results:**

Juniper AQLQ improved by 0.25 (95% confidence interval; −0.3, 0.8) units, overall asthma symptoms improved by 0.68 (0.1, 1.3) units, and rescue inhaler use decreased by 0.59 (−0.5, 1.6) daily administrations in azithromycin-treated compared to placebo-treated participants. Baseline IgA antibodies were positively associated with worsening overall asthma symptoms at follow-up (*p* = 0.04), but IgG was not (*p* = 0.63). Overall asthma symptom improvement attributable to azithromycin was 28% in high IgA participants versus 12% in low IgA participants (*p* for interaction = 0.27).

**Conclusions:**

Azithromycin did not improve Juniper AQLQ but appeared to improve overall asthma symptoms. Larger community-based trials of antichlamydial antibiotics for asthma are warranted.

## Editorial Commentary

**Background:** Chlamydia pneumoniae is a common bacterium thought to be responsible for a substantial proportion of community-acquired pneumonia and bronchitis infections. There is some observational evidence associating chronic C. pneumoniae infection with more severe symptoms in people with asthma. However, there are very little data from clinical trials determining whether treatment with antibiotics active against C. pneumoniae has an effect on the control of asthma.

**What this trial shows:** In this trial, the researchers randomized 45 adults who were being treated for asthma in primary care to receive either azithromycin (an antibiotic active against C. pneumoniae) or placebo, in addition to their usual asthma care. Participants were followed up for 3 mo after completion of treatment, during which time participants recorded data relating to their overall symptoms and daily activities on a 5-point scale, and use of bronchodilators. At the start of the trial, and at 3-mo follow-up, participants also completed a quality-of-life questionnaire using a validated scale. The primary objective of this trial was to investigate the feasibility of running an asthma trial in the primary care setting, and in using IVR telephone systems to collect the outcome data, reported in [13]. In this paper, the asthma outcomes are reported. Participants receiving azithromycin did not show a significant improvement in quality of life at 3-mo follow-up as compared to participants receiving placebo. However, the investigators did see a significant improvement in the overall symptoms recorded by participants receiving azithromycin, as compared to placebo.

**Strengths and limitations:** The randomization methods in the trial were appropriate, as was the choice of placebo as a comparison for azithromycin. However, the number of participants in the trial was small, and it is likely that many more participants would need to be recruited to conclusively demonstrate or disprove an effect of azithromycin on asthma-related quality of life. Further, the trial used three different measures for asthma outcomes: (1) the quality-of-life questionnaire, (2) measurement of symptoms and daily activities on a 5-point scale, and (3) bronchodilator use. Only the quality-of-life questionnaire is validated, making it difficult to compare the results with those of other asthma trials.

**Contribution to the evidence:** This trial provides suggestive evidence that azithromycin may have benefits in the treatment of asthma, but should not on its own lead to a change in practice. The study provides a good basis for a larger randomized trial of such treatments, which would need to assess reliably the effect of these drugs not only on symptoms but also on quality of life. Information gained from this trial would help to design several aspects of future studies, e.g., their size, follow-up duration, and suitable outcome measures.

## INTRODUCTION

*Chlamydia pneumoniae (C. pneumoniae)* is a ubiquitous intracellular human pathogen that is reported to cause approximately 10% of community-acquired pneumonia and 5% of acute bronchitis [1]. Chlamydial infections are characterized by persistence and immunopathologic damage to host target tissues, including the lung. C. pneumoniae infection has been associated with acute asthmatic bronchitis [2,3], bronchial hyperreactivity [4,5], new-onset asthma [6], chronic asthma [3,5], “infectious asthma” (asthma that first became symptomatic after an acute lower respiratory tract illness) [7], and asthma severity [8–10].

Chlamydia–asthma associations raise the question whether antibiotic treatment can improve asthma long term, but clinical trial evidence for persisting antichlamydial antibiotic effects on asthma is limited. An open-label before-after trial in 48 adults with stable, persistent asthma reported that over half of participants who were treated with 3–9 wk of antibiotics, consisting mostly of azithromycin, had major lasting clinical improvement or complete remission of asthma symptoms [11]. A preliminary report of a randomized trial of 10 d of telithromycin (a ketolide antibiotic with antichlamydial activity) in 278 adults with acute asthma exacerbations documented significant positive effects at the end of treatment [12] but did not report whether the improvement persisted.

The primary objectives of our pilot study were to investigate (1) the feasibility of performing an asthma clinical trial in practice settings and (2) the utility of an interactive voice-response (IVR) telephone system to collect asthma outcome data. The primary feasibility results have been published elsewhere [13]. In summary, we concluded that physician recruiting, randomizing, and completing a representative sample of adult asthma patients was feasible, but that utility of IVR in primary care research required further study, primarily because of underreporting. For example, 39 (87%) of 45 participants attended the follow-up clinical visit, 36 (80%) provided complete quality-of-life data, and 33 (73%) provided adequate asthma symptom and rescue bronchodilator medication data. The secondary objectives were to investigate (1) whether azithromycin treatment would affect any asthma outcome measures and (2) whether C. pneumoniae serology would be related to outcomes. This report presents the secondary results.

## METHODS

### Participants

We performed a community-based, multisite, randomized, allocation-concealed, blinded (patient, physician, data collector, data analyst), placebo-controlled trial in 45 adults with stable, persistent asthma recruited from primary care practices, an emergency room, and a community-based asthma research center. Potentially eligible patients were those aged 18 or older with a diagnosis of current asthma (variable symptoms of wheeze, chest tightness, cough, or shortness of breath triggered by a variety of stimuli) that was persistent, stable, and present for more than 3 mo prior to enrollment [14]. Stability was assessed during a 2–3 wk run-in period during which eligible patients remained in the same severity class (mild, moderate, or severe) and had no acute exacerbations. Documented objective evidence for reversible airway obstruction, either spontaneously or after treatment, was also required prior to randomization, either FEV1 change of 12% (and ≥200 mL) [14] or peak expiratory flow rate change of 25% (and ≥60 L/min) [15]. Exclusion criteria included (1) ingestion of any macrolide, tetracycline, or quinolone in the 6 wk before randomization, (2) macrolide allergy, (3) any unstable illness or other cause for symptoms, (4) use of coumadin, anticonvulsants, or digoxin and (5) pregnancy or lactation. The respective human subjects committees approved the study, and all participants provided written informed consent.

### Interventions

On the basis of the results of previous open-label treatment [11], we chose as study medication azithromycin, one 600-mg tablet daily for 3 consecutive days, followed by 600 mg weekly for an additional 5 wk (total dose 4,800 mg) or identical placebo tablets. All patients continued to receive usual care for asthma from their primary physician, who was blinded to treatment allocation.

### Objectives

The secondary results reported here are (1) azithromycin effects on asthma-specific quality of life (Juniper AQLQ), asthma symptoms, and rescue medication use, and (2) relationships of C. pneumoniae antibodies with these outcomes, and whether antibody levels were affected by treatment. Because this was a pilot study, we specified no primary asthma outcome.

### Asthma Clinical Outcomes

Participants completed the Juniper Asthma Quality of Life Questionnaire (Juniper AQLQ) [16] at baseline (pretreatment) and follow-up (3 mo after completion of treatment). Weekly throughout the study, patients were asked to record their symptoms, impairment in daily activities, and rescue bronchodilator use for the preceding 24-h period. Symptom categories were overall asthma symptoms, cough, wheeze, shortness of breath, and sleep disturbance due to asthma. Daily activity categories were impairment in work, housework, sports, and keeping appointments. Symptoms and activities were recorded using a 5-point scale (0 = none, no impairment; 1 = mild; 2 = moderate; 3 = severe; 4 = worst ever, could not perform). Rescue bronchodilator use was recorded as the number of episodes of use, not the total number of puffs. Juniper AQLQ and weekly diary data were reported by IVR, which did not accept incomplete reports.

### Serological Methods

Plasma samples were obtained at baseline and follow-up, frozen at −70 °C, and tested together in one batch. The serological ELISA testing was performed using a modification of our previously described method [17]. Briefly, Immunolon 2 plates (Dynex Technologies, Chantilly, Virginia, United States) were coated with 0.5 μg (based on protein concentration) of lysed C. pneumoniae AR39 elementary bodies in PBS for 48 h at 4 °C. After this period, plates were washed three times, and blocked for 90 min at 37 °C with PBS/3% ovalbumin (grade II)/0.1% Tween 20. Plates were then washed three times and incubated with triplicate samples of each patient's sera. For IgG measurement, sera were diluted 1:200 in PBS/0.1% ovalbumin (grade V)/0.05% Tween 20 and incubated for 1 h at 37 °C. For IgA measurement, sera were first diluted to 1:10 in GullSORB (Meridian Diagnostics Inc, Cincinnati, Ohio, United States), a reagent used for IgG antibody removal, to prevent interference. The final dilution of sera added to plates for IgA measurement was 1:50, and sera were incubated overnight at 4 °C [18]. Following incubation of sera, plates were washed three times, incubated with alkaline phosphatase-conjugated goat antihuman IgG or IgA (Jackson Immunoresearch Laboratories, West Grove, Pennsylvania, United States) for 30 min at 37 °C, and mixed with the substrate, *p*-nitrophenylphosphate (SigmaFAST tablets; Sigma Chemical Company, St. Louis, Missouri, United States). Absorbance was read as optical density (OD) at 405 nm. The OD value of a PBS-coated well that had no antigen (antigen-blank) was subtracted from the values for all test wells. Triplicate test OD values were averaged and reported for each patient. Laboratory personnel performing the ELISA test were blinded to clinical information on the patients. IgA measurements on the same samples tested on different days were highly correlated (*R* = 0.993, *p* < 0.0001, coefficient of variation <1%).

### Sample Size

Because no existing information on which to base sample size calculations was available, sample size was determined by available funding. We did not expect to observe any statistically significant treatment effects or serological associations.

### Randomization–Sequence Generation

At randomization, participants meeting final eligibility criteria were allocated to study medication bottles that were coded centrally using a computerized 1:1 (azithromycin:placebo) allocation ratio blocked by site. Block size was *n* = 6.

### Randomization–Allocation Concealment

Study physicians, research staff, participants, and data analysts were unaware of allocation due to central randomization and coding. Emergency unblinding envelopes were available, but study sites did not report opening any of them.

### Randomization–Implementation

An independent statistician, who had no further contact with study conduct, generated the randomization sequences. Bulk study medication tablets were bottled, labeled, and distributed by an independent pharmaceutical service that had no further role in study conduct.

### Statistical Methods

We employed the “intention to treat” principle. We did not impute values for missing data. We coded asthma outcomes so that positive values indicate improvement and negative values indicate worsening. For each subject, asthma outcome changes (change scores) were calculated as the absolute (arithmetic) difference between pre- and posttreatment values. For Juniper AQLQ, the change score was the difference between the baseline and follow-up values. For symptoms, activities, and bronchodilator use, the change score was the difference between (1) the mean of the first three weekly baseline values and (2) the mean of the last one to three available follow-up values obtained for study months 5 and 6. We also examined all available overall asthma symptom data aggregated by month and treatment group, and used linear regression to test for differences between azithromycin and placebo groups. We compared binary categorical variables using the Fisher exact test. We analyzed continuous, normally distributed outcomes using analysis of variance and controlled for subject characteristics using analysis of covariance. We performed a log transformation of OD prior to analysis. We performed Pearson correlation to examine relationships between continuous variables (e.g., between different asthma outcome scales and between pre- and posttreatment OD values) and linear regression to test for significance of the correlations. We calculated percentage changes in asthma symptom scores by dividing the appropriate raw change score by 5, the number of scale categories.

After determining that IgA OD was significantly associated with overall asthma symptom changes, we examined IgG and IgA levels as binary variables (low versus high antibody levels). Because this was a pilot study, no previous criteria were available. Therefore, cutoff values were determined a priori (before analysis) by inspection and defined as follows: high IgG was defined as an OD above the mean (1.18 OD units); high IgA was defined as an optical density greater than 0.5. In these analyses, we controlled for various baseline characteristics (including treatment allocation, with and without a treatment-antibody interaction term) as specified in each result. We report two-sided *p*-values <0.05 as significant. DataDesk® version 6 for Macintosh (DataDescription, Inc., Ithaca, New York, United States) was used to perform statistical calculations.

## RESULTS

### Participant Flow

**Figure 1:**
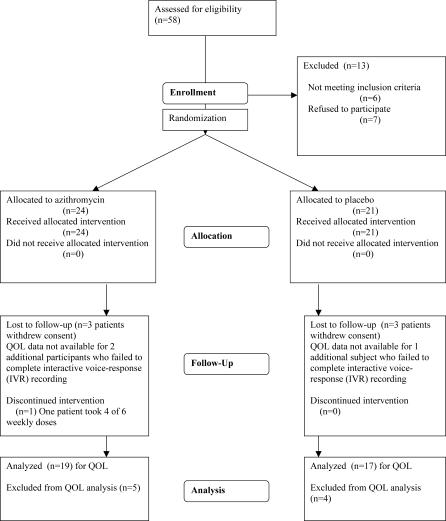
Study Flowchart

We recruited participants between September 1999 and December 2001. Forty-five participants meeting final eligibility criteria were randomized (Figure 1).

### Baseline Data

Most enrollees had mild to moderate persistent asthma, less than half reported an asthma exacerbation within the 2 y prior to enrollment, and none were oral steroid–dependent. The median duration of asthma symptoms prior to randomization was 14.5 y, and 44% reported that their initial asthma symptoms began after an acute respiratory illness, the so-called “infectious asthma” syndrome [19]. With the exception of sex distribution, the randomized groups were well balanced on baseline characteristics (Table 1).

**Table 1:**
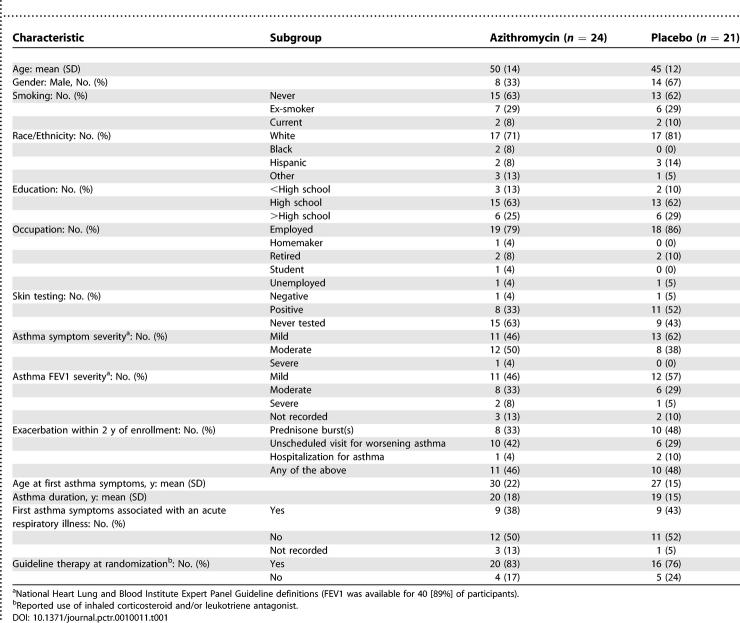
Baseline Patient Characteristics

Study medication adherence was complete except for one azithromycin subject who reported taking only four of six weekly doses. During usual care, two azithromycin and three placebo participants received standard (7–10 d) courses of antibiotics, only one of which had antichlamydial activity. One azithromycin and two placebo participants were prescribed increased doses of anti-inflammatory controller medication. Five azithromycin and two placebo participants reported mild/moderate gastrointestinal side effects. A single serious adverse event was reported: one subject allocated placebo died of asthma-related causes.

### Results of Blinding

Three weeks after treatment began, 40 (89%) of 45 randomized participants provided information about results of blinding. Five of 20 participants allocated to azithromycin and four of 20 allocated to placebo guessed they were taking azithromycin, seven in the azithromycin group and eight in the placebo group guessed they were taking placebo, and seven and eight, respectively, indicated that they were not sure whether they were taking azithromycin or placebo (*p* = 0.9, chi-square test).

### Outcomes and Estimation

#### Asthma outcomes.

**Figure 2:**
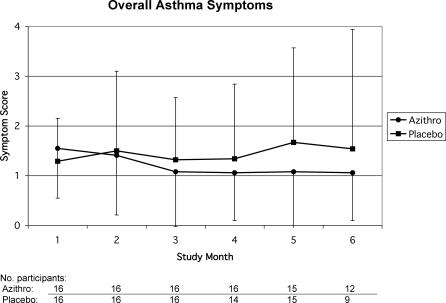
Overall Asthma Symptoms in the Azithromycin and Placebo Groups Months 1 and 2: Baseline and treatment period. Month 3: Completion of treatment. Months 4–6: Posttreatment. Numbers of participants included in each data point are indicated under the *x*-axis. Error bars represent 95% confidence intervals for individual data points. Linear regression analysis showed a significant (*p* = 0.04) overall difference between azithromycin and placebo group patterns.

Table 2 presents results for asthma quality of life (Juniper AQLQ), symptoms, daily function, and bronchodilator use. Juniper AQLQ improved by +0.25 units (−0.35, 0.84), and bronchodilator use episodes improved by +0.59 (−0.47, 1.64) daily administrations. Differences favored azithromycin for all asthma symptom and activity outcomes except work (−0.02 units). Overall asthma symptoms improved in the azithromycin group (+0.55) and worsened in the placebo group (−0.13). The effect size (+0.68; 0.05, 1.29) in favor of azithromycin was statistically significant (*p* = 0.04) and remained significant (*p* = 0.004) after controlling for antibody level, age, sex, smoking, skin test status, infectious onset, age of asthma onset, asthma severity, and change in controller medication use (mostly inhaled corticosteroids). Figure 2 presents overall asthma symptom patterns by study month according to treatment allocation. Maximum overall symptom improvement in the azithromycin group appeared by 3 mo, remained stable through 6 mo, and was significantly (*p* = 0.04) different from placebo by linear regression analysis.

**Table 2:**
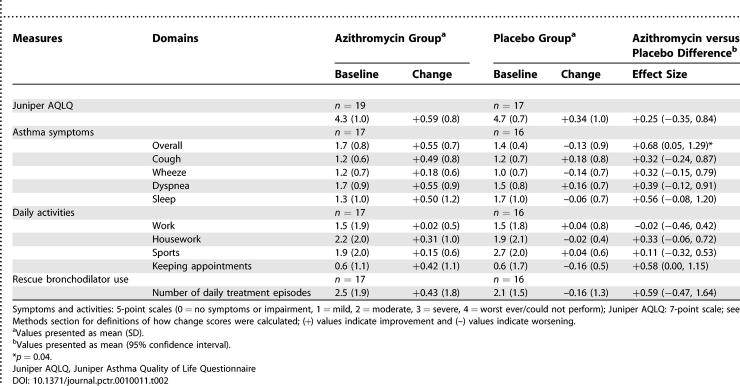
Juniper AQLQ, Asthma Symptoms, Daily Activities, and Rescue Bronchodilator Use: Baseline Values and Changes at 3 Mo after Finishing Study Medication

Neither Juniper AQLQ nor the Juniper AQLQ symptom subscale change scores correlated with overall asthma symptom change scores (*r* = 0.19, *p* = 0.31; *r* = 0.16, *p* = 0.38, respectively) or with bronchodilator change scores (*r* = 0.27, *p* = 0.14; *r* = 0.31, *p* = .08). Overall asthma symptom change scores were, however, significantly correlated with bronchodilator changes (*r* = 0.55, *p* < 0.001).

#### Serological results.

**Figure 3:**
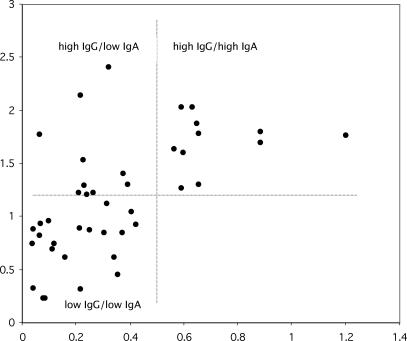
Scatter Plot of Chlamydia pneumoniae–specific IgG and IgA Optical Density (OD) Values Obtained on Available Baseline Patient Sera (*n* = 42) *Y-axis:* IgG OD; *X-axis:* IgA OD. High IgG was defined by inspection as an OD above the mean (1.18 OD units). High IgA was defined by inspection as an OD greater than 0.5.

Baseline serological samples were available for 43 (96%) participants. Because treatment was significantly related to overall asthma symptoms (see “Asthma outcomes” section above) serological analyses were controlled for treatment allocation. Baseline serum IgA ELISA OD, as a continuous variable, was positively and significantly associated (*p* = 0.04) with overall asthma symptom changes, i.e., higher serological values predicted worsening asthma at follow-up. Baseline IgG OD was not associated with symptom changes (*p* = 0.63). We then explored antibody levels as binary variables. An IgG/IgA scatter plot revealed that participants were distributed among three of the four possible categories (high IgG/high IgA [*n* = 11], high IgG/low IgA [*n* = 10], and low IgG/low IgA [*n* = 21]). There were no participants in the low IgG/high IgA category (Figure 3). High IgA antibody level category (*p* = 0.03), but not high IgG (*p* = 0.34), was associated with worsening overall asthma symptoms at follow-up. Results remained significant (*p* = 0.04) when gender was included as an additional independent variable.

Thirty participants had baseline IgA results and overall asthma symptom data at follow-up. The overall asthma symptom effect size favoring azithromycin was 28% (13%, 42%) in high IgA participants (*p* = 0.003) versus 12% (−3%, 29%) in low IgA participants (*p* = 0.14). A treatment-antibody interaction term was not significant (*p* = 0.27). Placebo-treated participants with high IgA levels (*n* = 3) had a worsening overall asthma symptom score of −1.2 (−2.0, −0.5) units. Placebo-treated participants with low IgA levels (*n* = 11) had an improvement of +0.1 (−1.6, 1.7) units. Azithromycin-treated participants with high IgA (*n* = 6) had an improvement of +0.2 (−0.8, 1.2) units, and azithromycin-treated participants with low IgA (*n* = 10) had improvement of 0.7 (−1.4, 2.8) units. The correlation between baseline and follow-up IgA antibody ODs was 0.967 (*p* < 0.0001) and did not differ by treatment allocation (*p* = 0.62).

## DISCUSSION

### Interpretation

#### Asthma outcomes.

We did not find significant improvements in Juniper AQLQ resulting from azithromycin treatment. A post-hoc power calculation (α = 0.05, β = 0.80, pooled SD = 0.89) indicated that 400 completed participants would be needed to detect the difference we found in Juniper AQLQ (0.25 units). The Juniper AQLQ confidence intervals included 0.5 unit, which is considered the minimum important change [20]. Detecting this difference, if it existed, would require 100 completed participants. An overall difference of one unit in Juniper AQLQ is outside the confidence limits of our results, but might be plausible for participants with more severe disease than we studied. Larger trials, with longer follow-up periods and preferably in patients with more severe asthma, are warranted to determine whether azithromycin can improve asthma-specific quality of life and medication use, as well as asthma symptoms. Ideally, such trials should include microbiological assessment for atypical infections, with the caveat that current diagnostic techniques are indirect and have not been validated for chronic lung infection, or for microbiologic eradication.

Neither the Juniper AQLQ nor the Juniper AQLQ symptom subscale correlated significantly with overall asthma symptom changes or with bronchodilator changes in our study, whereas overall asthma symptom changes and bronchodilator use changes were significantly correlated with each other. The findings for the Juniper AQLQ are consistent with the results of factor analysis of asthma outcome data from three asthma clinical trials [21]. That analysis found that Juniper AQLQ factored independently from symptoms and medication use, and that symptoms and medication use factored together. In our study, the Juniper AQLQ symptom subscale did not correlate with either the overall asthma symptom or bronchodilator measures. Evidence favors the concept that asthma outcomes occupy separate domains, although debate surrounds their exact number and description [21,22]. The Juniper AQLQ is designed to capture a 2-wk look-back interval, whereas our overall asthma symptom and bronchodilator scores measured the previous 24 h. The lack of correlation we found between the Juniper AQLQ symptom subscale changes and the overall asthma symptom changes suggests that the “look-back” duration may be an important variable in determining the results of asthma domain factor analyses.

We found suggestive evidence that adjunctive azithromycin treatment had persisting benefit on overall asthma symptoms, as measured by our previously unvalidated 5-point symptom scale. The overall asthma symptom changes, as measured by this 5-point scale, correlated significantly with patient-reported bronchodilator use changes, thus providing limited support for its validity. The apparent improvement in overall asthma was maximal by the third study month and persisted until final follow-up 3 mo after treatment completion. This result is consistent with a previous open-label before-after study in which antibiotic (mainly azithromycin) responders reported that maximum improvement occurred by the third study month and persisted after treatment ended [11]. However, these results must be interpreted cautiously since they were obtained in a pilot study that did not specify a primary asthma outcome. Nevertheless, these preliminary results support performing further clinical trials in larger representative samples of asthma patients followed for longer time periods.

#### Serological results.

A growing body of evidence from clinical observations and case-control studies supports an association of C. pneumoniae infection and asthma [23]. Results from the study reported here show that C. pneumoniae–specific IgA antibodies predict 6-mo asthma prognosis, as measured by an overall asthma symptom scale. This relationship did not hold for IgG antibodies. There are no validated serodiagnostic tests for chronic C. pneumoniae lung infection. C. pneumoniae–specific IgA antibodies have been suggested as potential seroepidemiological markers for chronic infection in asthma [7] and might be useful in epidemiological and primary care asthma studies where direct microbiologic sampling of the lower airway is impractical. The microimmunofluorescence (MIF) test is the accepted “gold standard” for C. pneumoniae serologic testing [24]. Our study employed an ELISA test with proven excellent reproducibility that we previously validated against the MIF test [25]. Our scatter plot results (Figure 3) are also consistent with previous MIF data indicating that almost all patients with detectable C. pneumoniae–specific IgA also had detectable high titers of IgG [26]. OD reproducibility, validation against, and consistency with previous MIF results support the use of our ELISA. As part of the ELISA method for measuring IgA antibodies, we performed overnight incubation of patient samples with the aim of improving assay sensitivity, as suggested by the work of others using the MIF test [18]. Our results furnish further evidence [7] that C. pneumoniae–specific IgA may have value as a seroepidemiological tool in primary care–based asthma studies.

Since the half-life of IgA is short (about 1 wk [27]), persisting levels of IgA may indicate ongoing antigenic stimulation, e.g., chronic infection. In acute, uncomplicated genitourinary infection, loss of organism-specific IgA can correlate with microbiologic eradication of Chlamydia trachomatis [28]. It is well recognized that eradication of chronic chlamydial infections are problematic, and we were unable to demonstrate any significant decrease in C. pneumoniae–specific IgA after 6 wk of treatment with azithromycin. We suggest measuring IgA antibodies after longer treatment courses.

Symptom improvement attributable to azithromycin was greater (+1.4, or 28%) in the high IgA category than in the low IgA category (+0.6, or 12%), suggesting that IgA might be useful in predicting treatment response. A treatment-antibody interaction term was not statistically significant (*p* = 0.27). Further studies are required to investigate whether chlamydial biomarkers predict treatment outcome.

### Generalizability

We enrolled a representative sample of community-based, nonreferred adults with mainly mild to moderate persistent stable asthma. We did not exclude participants on the basis of smoking or on the basis of an element of coexisting fixed obstruction, since these characteristics are present in about half of adult asthma patients in the community [29]. Our results might not apply to asthma patients with milder symptoms, or to those with more severe disease (e.g., those who are steroid-dependent or experiencing frequent exacerbations).

### Overall Evidence

A recently updated Cochrane Review concluded that there is insufficient evidence to support or refute the use of macrolides in patients with chronic asthma, and that further studies are needed to define the potential role of macrolides in asthmatic patients with chronic atypical infections [30]. Our trial provides evidence relating to the role of the azalide macrolide, azithromycin, in the treatment of asthma over a 3-mo follow-up period. Results for some of our outcome data (overall asthma symptoms) provide suggestive evidence for a treatment effect over that period of time. Some clinicians have prescribed macrolides as adjunctive treatment for asthma since the 1950s [31], believing that macrolides confer benefit via direct anti-inflammatory mechanisms of action and/or other nonantibiotic effects [32]. An acknowledged characteristic of anti-inflammatory mechanisms is the requirement for ongoing administration of the anti-inflammatory medication to maintain clinical effectiveness [33]. If macrolides benefit asthma via antibiotic mechanisms of action, then continued clinical benefits after completion of treatment could be expected.

### Limitations

Limitations of this study include its pilot nature, limited size and array of outcome measures, and relatively short duration. Randomization failed to achieve gender balance, but results remained significant after adjusting for gender. The outcome measures (Juniper AQLQ, symptoms, and bronchodilator use) primarily reflect asthma control in our study population with mild to moderately severe asthma. We did not assess effects on asthma exacerbations that are an additional important outcome. Strengths include the prospective clinical trial study design, representative sample of community-based patients, requirement for objective pulmonary function evidence to support the asthma diagnosis, use of a validated serologic technique, and analysis of patient-oriented outcomes. Our results support performance of further studies to investigate whether asthma is treatable with antibiotics.

## SUPPORTING INFORMATION

CONSORT Checklist Click here for additional data file. (50 KB DOC)

Trial Protocol Click here for additional data file. (30 KB DOC)

## Abbreviations

IVR: interactive voice-response
Juniper AQLQ: Juniper Asthma Quality of Life Questionnaire
MIF test: microimmunofluorescence test
OD: optical density
