# Supplementary material for: Secondary Outcomes of a Pilot Randomized Trial of Azithromycin Treatment for Asthma
Source: PLoS Clin Trials. 2006 Jun 30;1(2):e11. doi: 10.1371/journal.pctr.0010011 (PMC1488900; doi:10.1371/journal.pctr.0010011)
Supplement: Trial Protocol [file pctr.0010011.sd002.doc]

HYPOTHESIS 

The hypothesis to be tested is that a randomized, controlled treatment trial of antibiotic treatment for adult asthma
can be carried out successfully in a geographically dispersed
practice-based research network.

The secondary hypothesis is that trends favoring antibiotic treatment will emerge
during followup, allowing estimation of sample size(s) for an adequately powered study protocol to be submitted to the National Institutes of Health (NIH) as an RO-1 grant.

        ABSTRACT

Asthma is a chronic inflammatory bronchial condition of unknown
etiology.  Decades ago many clinicians believed that infection played a
major role in asthma etiology, but current expert opinion favors the view
that asthma is a noninfectious condition whose root cause is
inflammation.  Therefore chronic antiinflammatory therapy, mainly inhaled
corticosteroids, is currently advocated as primary anti-asthma treatment. 
It is important to recognize, however, that antiinflammatory therapy is
palliative, not curative.

A growing body of evidence implicates chronic bronchial infection with
Chlamydia pneumoniae in the pathogenesis of asthma in both adults and
children.  Organism identification studies (culture and PCR) suggest that
up to one-half of children with asthma may be chronically infected by C.
pneumoniae, and seroepidemiologic studies in adults are consistent with
chronic C. pneumoniae infection in the majority of adult-onset asthmatics.
 Furthermore, case reports and uncontrolled trials have provided
provocative but inconclusive evidence that treatment of C. pneumoniae
infection in both children and adults with asthma can favorably affect the
natural history of this disorder.

We propose a randomized, placebo-controlled, triple-blinded study of
antichlamydial antimicrobial therapy in adult asthma.  Results will
help to determine whether antimicrobial therapy is effective in treating
some adult asthma syndromes.  Positive results would have significant
public health implications.  Methodologies developed for use in this trial
may expedite future studies in practice-based research networks.

BACKGROUND AND METHODS 

1.  BACKGROUND AND PURPOSE  Asthma is a chronic inflammatory lung
condition of unknown etiology.  The cost for asthma care in the US has
been conservatively estimated at over $6 billion (1990 data) with 55% of
costs attributable to asthma in patients 18 years or older.1  Currently
recommended antiinflammatory therapies are palliative, not curative2 and
asthma morbidity and mortality appears to be increasing in recent
decades.3

An emerging body of evidence suggests that chronic Chlamydia
pneumoniae respiratory tract infection may be responsible for the
bronchial inflammation underlying some asthma syndromes,4 including
adult-onset asthma.5  Preliminary reports of complete asthma remission
following prolonged treatment with macrolides and tetracyclines
(effective against chlamydia) raise the possibility of "cure" for some
patients with the infectious asthma syndrome.6  An analogy with
Helicobacter pylori infection as a cause of peptic ulcer disease is
suggested.  Our primary goal is to test the feasibility
of performing a randomized antibiotic treatment trial in a geographically dispersed
practice-based research network (PBRN) where the majority of asthma
is encountered and managed.1  Our secondary goal is to determine in this controlled trial whether
addition of antibiotic treatment to usual care for adult-onset asthma is
superior to placebo.

PRIMARY OBJECTIVE  To test the feasibility of implementing the trial
in a practice-based research network.  The specific feasibility questions
to be addressed are: (1) Can the PBRN practices successfully recruit
and enroll eligible participants? (2) Can the central coordinating site
develop and maintain practice interest? (3) Can patient-reported data
collection be facilitated by use of an interactive voice-response (IVR)
telephone system, thus relieving the PBRN practices of responsibility for
most data collection and management?

SECONDARY OBJECTIVE  To perform a randomized, triple-blinded
(physician, patient and data analyst) controlled trial of antibiotic treatment
for adult asthma.

PREVIOUS AND RELATED WORK  The PI for this project has published
the majority of articles associating asthma with chronic chlamydial
infection.4

His first major publication to describe this association (partially funded by
the AAFP) was successfully performed in non-academic primary care
clinics from a single geographic area.5  The PI has also successfully
performed a study describing adult-onset asthma prevalence with the
active cooperation of 60 geographically dispersed PBRN practices.7  A
study currently recruiting adult asthma patients for a randomized trial of
macrolide treatment in New Zealand, Australia and Argentina has
confirmed that 60% of 459 adults with asthma (mixture of adult- and
childhood-onset) have antibody levels indicative of possible chronic
infection with Chlamydia pneumoniae (Francesco Blasi: personal
communication).  Thus it is important to proceed with randomized trials in
North America, since results of several trials might have a significant
effect on asthma management worldwide.

METHODS  
1. Patient selection criteria: Adults (ages 18 and older) with stable
chronic (>3 months duration) persistent asthma as defined by the
updated Expert Panel Report (EPR2)2 are eligible for enrollment. 
Patients will not be enrolled during an asthma exacerbation.  Stability of
asthma must be demonstrated during a two-week (pre-randomization)
run-in period.

2. Study setting: Primary care (mostly family practice) clinicians in
Wisconsin and elsewhere in North America were recruited from (1) the
Wisconsin Research Network (WReN), (2) participants in a previous
asthma prevalence study7 and (3) participants from an interest group at
the 1997 ASPN Annual Convocation of Practices.  See Appendix 1 for a
list of study participants.  Clinics in an urban area will be included to
increase representation of indigent non-white asthma patients.

3. Sample size: We plan to accrue 100 evaluable patients in this pilot
study.  We expect to enroll at least 6 eligible patients per site from 20
sites (20% dropout rate).  We estimate that the incidence of adult-onset
asthma is 1/1000/year8 and that the average primary care practitioner
cares for 1000 adults (a conservative estimate).  Therefore, a five
person primary care group practice will have in their patient population
approximately 50 potentially eligible adult-onset asthma patients whose
disease duration is ten years or less in addition to a greater number of adults with asthma that began in childhood.

The sample size for this pilot was determined by feasibility issues, e.g.,
by an estimate of the number of patients we can afford to enroll with the
limited funds available.  Adult-onset asthma patients are more likely to exhibit serologic
evidence of chronic C. pneumoniae infection than patients with a history
of childhood-onset asthma.4  A detailed but speculative power analysis
(available by request) suggests that a total sample size in the range of
several hundred patients is desirable.  This estimate depends on several
variables including the C. pneumoniae seroprevalence in the target
population, the duration of symptomatic disease prior to treatment and the
amount of fixed obstruction accompanying the asthma.6

4. Study design: Randomized, placebo-controlled trial of azithromycin
(600 mg orally, daily for 3 days, then once per week for an additional 5 weeks (total dose 4800 mg) v. matching placebo
added to usual care for asthma.  The antibiotic regimen was chosen
based on the results of a previous open-label study demonstrating
persistent improvement in asthma after azithromycin therapy.6  Usual care is
defined as the care usually provided to asthma patients at the study site. 
Efficacy will be determined at 3 months post-treatment completion.

5. Outcome measures: Patient-reported measures include (1) a generic
and a disease-specific quality-of-life (QOL) questionnaire and (2) asthma
diary data, including symptoms, function, bronchodilator use and diurnal
peak expiratory flow rate (PEFR).  Clinician-derived measures include (1)
clinician assessments and (2) pre-bronchodilator FEV1.  The generic
QOL questionnaire will be the SF-36 which has been validated for
asthma.9  The disease-specific instrument of Juniper et. al. has been
extensively used in prior asthma research and will be employed in this
study.10  The patient diary incorporates standard measures of asthma
symptoms, bronchodilator use and PEFR used in previous studies, as
well as validated daily function measures that may supplement SF-36
information11 (Appendix 2).  The primary outcome measure will be
difference (azithromycin v. placebo) in pre-bronchodilator FEV1 at the
3-month post-treatment visit.  The secondary measures will be
differences in bronchodilator use, clinical status (exacerbations requiring
steroid administration, emergency facility use and/or hospitalization) and
QOL.  Outcomes will be post-stratified on evidence/no evidence of C.
pneumoniae infection.  Evidence of infection is defined as a positive PCR in peripheral blood mononuclear cells
and/or an IgG titer of 1:64 or greater and/or an IgA titer of 1:16 or
greater.

6. Visit schedule: Five visits over 5 months are planned: visit 1 (entry
visit, week -2) for initial eligibility assessment and informed consent; visit
2 (randomization, week 0) for final eligibility assessment, pre-treatment
clinical results, pre-bronchodilator FEV1 and chlamydia studies, and
dispensing of study medication; visit 3 (mid-therapy, week 3) to assess
initial compliance and side effects; visit 4 (end-therapy, week 6) to
assess final compliance and side effects; and visit 5 
(follow-up, week 18, 3 months after finishing study treatment) to record
post-treatment clinical results, pre-bronchodilator FEV1 and chlamydia
studies.  Clinical results will include (1) history of exacerbations requiring
steroid administration, emergency facility use and/or hospitalization, (2) changes in bronchodilator and other asthma medication use and (3)
global assessment of asthma severity score validated in a previous
study.6  Chlamydia studies will include C. pneumoniae-specific (1)
polymerase chain reaction (PCR) testing of peripheral blood mononuclear cells and
(2) serology (IgG and IgA).

7.  Interactive voice-response system: Interactive voice response
telephone systems have become a routine part of daily life for most
Americans who make airline reservations or call banks and other
businesses including medical offices.  Sophisticated IVR systems to
diagnose and manage psychiatric conditions, including depression and
obsessive-compulsive disorder, have been developed and tested at the
Dean Foundation for Health Research and Education, Madison,
Wisconsin.12  The generic software drivers used in the existing
applications can be readily adapted for use in data collection for asthma
studies (Appendix 3).  All patient-reported data (QOL questionnaires and
diary information) will be recorded directly by patients into the IVR
system databank using a toll-free 800 number.  The IVR system has
several advantages over traditional data collection methods: (1) data is
entered directly into digital format, avoiding data entry errors, (2)
practices are not burdened by data collection tasks, (3) patient
compliance can be monitored systematically, (4) patients can dial in from
anywhere in the world, and (5) previous experience indicates that
patients offer as much or more information to computerized IVR systems
as they do during face to face interviews with clinicians.12

8. Statistical testing:  Between group comparisons (active drug v.
placebo) will be performed using an unpaired t-test for normally
distributed continuous data and the nonparametric Mann-Whitney U test
for non-normally distributed ranked data.  Within group differences
(followup compared to baseline) will be performed using the paired t-test
for normally distributed continuous data and the Wilcoxon signed rank
test for non-normally distributed ranked data.  Multivariate modelling of
normally distributed continuous dependent variables (to control for
discrete and/or continuous covariates as needed) will be performed
using ANOVA and/or ANOCOVA.  Logistic regression will be used to
control for confounding when the dependent variable of interest is a
noncontinuous bivariate.  Other tests, including Fishers exact test for 2 x
2 tables and the chi-square test for n x r tables, will be used when
appropriate.

9. Study flow diagram

Appendix 4 contains details of the ASTHMA TRIAL pilot study protocol,
including an executive summary, detailed eligibility and exclusion criteria,
a summary of duties for patients, study physicians, on-site coordinators
and the central site study manager, the EPR2 definitions for persistent
asthma and the study protocol reproduced in tabular format.

TIMELINE  The majority of the patients will be enrolled, randomized and completed within 12 months after beginning enrollment.  Thus, feasibility results will be available and reported upon within a one year time period.

We anticipate patient enrollment to be completed over a 12 to 18
month time period.  With 6 months allowed for follow-up and database
completion, non-post-stratified study results will be available 18 to 24
months after study initiation.  Time of availability of results post-stratified
on presence/absence of chlamydia infection will depend on the
reference laboratory but testing is expected to be done soon after study
completion.

WHO WILL BENEFIT?  Positive results of randomized trials of antibiotics in
asthma will change asthma treatment as peptic ulcer disease treatment
was changed after the demonstration that antibiotics could cure ulcers. 
The results of this trial will be presented at national and international
meetings and submitted for publication to a major general medical journal.


FOLLOWUP STUDIES PLANNED  If this study is successful we plan (1) to use the results in support of an RO-1 grant to NIH for an adequately powered study based on the results of this pilot and (2) to
monitor the study cohort long-term, including the outcome of offering
active treatment to the placebo group if results confirm the hypothesis of
a positive treatment effect.

REFERENCE SECTION

1.Weiss KB, Gergen PJ, Hodgson T. An economic evaluation of asthma in
the United States. New Engl J Med 1992; 326:862-866.

2.Expert Panel Report II. Guidelines for the diagnosis and management of
asthma. US Department of Health and Human Services. Public Health
Service.  National Institutes of Health, National Heart, Lung, and Blood
Institute.  February 1997.

3.Burr ML. Is asthma increasing? J Epidemiol Comm Health 1987;
41:185-189.

4.Hahn DL. Intracellular pathogens and their role in asthma: Chlamydia
pneumoniae in adult patients. Eur Respir Rev 1996; 6:224-230.

5.Hahn DL, Dodge R, Golubjatnikov R. Association of Chlamydia
pneumoniae (strain TWAR) infection with wheezing, asthmatic bronchitis
and adult-onset asthma. JAMA 1991; 266:225-230.

6.Hahn DL. Treatment of Chlamydia pneumoniae infection in adult asthma:
a before-after trial. J Fam Pract 1995; 41:345-351.

7.Hahn DL, Beasley JW. Diagnosed and possible undiagnosed asthma: a
Wisconsin Research Network (WReN) study. J Fam Pract 1994;
38:373-379.

8.Dodge RR, Burrows B. The prevalence and incidence of asthma and
asthma-like symptoms in a general population sample. Am J Resp Dis
1980;122:567-575.

9.Bousquet J, Knani J, Dhivert H, et al. Quality-of-life in asthma I. Internal
consistency and validity of the SF-36 questionnaire. Am J Respir Crit
Care Med 1994; 149:371-375.

10.Juniper EF, Guyatt GH. Evaluation of impairment of health-related
quality of life in asthma; development of a questionnaire for use in clinical
trials. Thorax 1992; 47:76-83.

11.Hyland ME, Crocker GR. Validation of an asthma quality of life diary in
a clinical trial. Thorax 1995; 50:724-730.

12.Kobak KA, Taylor LvH, Dottl SL, et al. A computer-administered
telephone interview to identify mental disorders. JAMA 1997;
278:905-910.
